# Ubiquitous presence of piscidin-1 in Atlantic cod as evidenced by immunolocalisation

**DOI:** 10.1186/1746-6148-8-46

**Published:** 2012-07-11

**Authors:** Jareeporn Ruangsri, Jorge M O Fernandes, Jan H W M Rombout, Monica F Brinchmann, Viswanath Kiron

**Affiliations:** 1 Faculty of Biosciences and Aquaculture, University of Nordland, 8049, Bodø, Norway; 2 Cell Biology and Immunology Group, Wageningen Institute of Animal Sciences, Wageningen University, Marijkeweg 40, 6709 PG, Wageningen, The Netherlands

**Keywords:** Piscidin, Antimicrobial peptide, Innate immunity, Multi-functionality, *Gadus morhua*

## Abstract

****Background**:**

Antimicrobial peptides (AMPs), the natural antibiotics bestowed upon all forms of life, consist of small molecular weight proteins with a broad spectrum antimicrobial activity against a variety of pathogenic microorganisms. Piscidins are one of the AMP families that are imperative for the innate defence mechanisms of teleosts. Atlantic cod, a basal fish belonging to the superorder Paracanthopterygii also possesses multiple piscidin peptides. Two piscidin paralogues (*pis1* and *pis2*) and a novel alternative splice variant of *pis2* of this fish were previously described by us. To shed light on other potent roles of these molecules, now we have mapped the distribution of piscidin 1 (Pis1), in different tissues and organs of cod through immunohistochemistry (IHC) employing an affinity purified polyclonal antibody specific to Pis1.

****Results**:**

Various cell types and tissues of Atlantic cod including those from the immune organs of naïve fish are armed with Pis1 peptide. Different types of the blood leucocytes and phagocytic cells among the leucocytes examined gave a relatively strong indication of Pis1 immunopositivity. In addition, other cell types such as hematopoietic cells, epithelial cells and multi-granular cells located in the mucosal and hematopoietic tissues were also Pis1-immunoreactive. More interestingly, chondrocytes appear to produce Pis1 and this is the first report on the presence of an AMP in cartilage tissue of fish. Furthermore, Pis1 immunopositivity was detected in other tissues and organs of naïve fish including neural tissues, exocrine and endocrine glands, compound gland cells, excretory kidney, intestinal and respiratory epithelial cells, swim bladder, skin and hypodermis layer, myosepta, liver, heart, eye and oocytes.

**Conclusions:**

Pis1 peptide is produced by various cell types located in different tissues and organs of Atlantic cod. It is present in all immune-related organs of naïve fish and the elevated peptide expression following phagocytosis strongly suggest their involvement in innate defence. Further, its widespread occurrence in non-immune tissues and organs of apparently healthy fish implies that piscidin may have other functions in addition to its role as an immune effector molecule.

## **Background**

Atlantic cod (*Gadus morhua* L.) is a demersal fish that is widely distributed in the North Atlantic region, the Baltic Sea and the Barents Sea. Commercial production of this fish has been undertaken mainly by Norway, though fraught with several challenges. There has been great interest in understanding the immune system of this fish species. It has been confirmed recently that cod has a unique immune architecture compared to other vertebrates as they are devoid of genes for major histocompatibility complex (MHC) II, cluster of differentiation 4 (CD4) and invariant chain (Ii) [1]; all of them are attributed to a normal functioning of adaptive immunity. Earlier studies [2-4] that examined the antibody responses of Atlantic cod have revealed that cod relies more on innate than adaptive defence mechanisms. On the other hand, cod exhibits an incredible ability to defend itself against pathogens [5]. The effective functioning of the innate immune system could be due to the presence of a number of MHC I loci and the unique organization of Toll-like receptor (TLR) families in the genome [1,6]. Furthermore, our contribution to the knowledge on the innate immune components of Atlantic cod is that several tissues of the fish are armed with a battery of peptides with antimicrobial activity [7].

Antimicrobial peptides (AMPs), the natural antibiotics bestowed upon all forms of life, consist of small molecular weight proteins with a broad spectrum antimicrobial activity against a variety of pathogenic microorganisms [8]. Several fish AMPs, are described as essential innate defence molecules [9,10]. Piscidins are one of the most potent AMPs found in both freshwater and marine teleosts [11-17] and their antimicrobial properties enable them to inhibit the growth of bacteria, fungi, viruses and parasites [14,18-21]. Immunohistochemical studies have shown that various cell types in different tissues and organs, particularly the interface tissues that are in constant interaction with the environment (e.g. gills, skin, alimentary tract) and the hematopoietic tissues are involved in the production of piscidin peptides [13,14,22,23].

Recently two piscidin paralogues (*pis1* and *pis2*) and a novel alternative splice variant of piscidin (*pis2b*) [17,24] were described for cod. Moreover, we have reported that *pis* genes of Atlantic cod have undergone structural diversifications through positive selection [17]. Our additional studies have shed light on the variation in their gene expressions in different tissues of adult fish and during developmental stages and on the broad antibacterial properties of the synthetic peptides of Atlantic cod piscidin [24] . To further understand the potent role of piscidin peptides in Atlantic cod, immunohistochemistry (IHC) was used to identify tissue and cell distribution of Pis1, using an anti-Pis1 antibody.

## **Methods**

### **Anti-Pis1 antibody**

Affinity-purified rabbit polyclonal anti-Pis1 antibody raised against the whole mature peptide sequence of Atlantic cod Pis1, prepared on demand (GenScript, New Jersey, USA) was used in the present study. The peptide antigen corresponding to C-FIHHIIGWISHGVRAIHR AIHG was used later for polyclonal antibody production following the manufacturer’s procedures (GenScript). Briefly, synthetic peptide was conjugated to keyhole limpet hemocyanin (KLH) and then injected into rabbits. The antiserum was then affinity purified by running over a column having the 23-mer Pis1 fragment conjugated to cyanogen bromide-activated agarose as an immunosorbent. The resulting titer of the affinity-purified antibody was 1:64,000, confirmed by ELISA. The peptide-specific antibody had less than 1 % cross-reactivity in the ELISA, where 1 % cross-reactivity is 100 times more antibody than is required to produce the same optical density with either free KLH, conjugated KLH, or free peptide that shares less than three amino acids in the sequence (according to GenScript). Furthermore, through Western blot analysis it was determined that anti-Pis1 antibody did not react with either synthetic Pis2 or Pis2b peptide (1 μg of Pis2; FLHHIVGLIHHGLSLFGDRAD or Pis2b; FLHHIVGLIHHGKLDMYRSNN) of Atlantic cod, while its immunoreactivity was strong with 1 μg of Pis1 peptide Additional file ( 1: Figure S1).

### **Fish and their maintenance**

Apparently healthy hatchery produced Atlantic cod were maintained in the indoor facilities at the research station of University of Nordland, Bodø, Norway. The fish (200‐300 g) selected for the study were from among those reared at 7‐8°C in 2800 l fiberglass tanks and fed daily on a commercial feed (Amber Neptun; Skretting AS, Norway). All animal handling protocols were approved by the National Animal Research Authority (Forsøksdyrutvalget, FDU; id numbers: 2453 and 3207) in Norway.

### **Leucocyte preparation and fixation**

Experimental fish were anesthetized using a sub-lethal dose (100 mg·l^−1^) of tricaine methanesulphonate (MS-222, Argent Chemical Laboratories, Washington, USA) and immediately euthanised. Blood was collected from the caudal vein using a heparinised (Heparin 150 Unit⋅ml^−1^, Sigma, Missouri, USA) syringe. Following aseptic procedures, freshly dissected head kidney and spleen were put in L-15 medium (Leibovitz, Sigma) containing 2 % fetal bovine serum (Sigma) and 100 U⋅ml^−1^ heparin. Subsequently, the tissues were placed on a 100 μm filter and teased gently with a sterile syringe plunger to prepare cell suspensions in the medium contained in a Petri dish. The cells collected were transferred to a 15 ml sterile tube. In order to achieve a good quality harvest of leucocytes from the peripheral blood, head kidney or spleen cells, their cell suspensions were centrifuged at 1000 *g* for 5 min at 4°C. Thereafter the buffy coat of leucocytes was put into a new 1.5 ml sterile tube and was resuspended in L-15 medium to get an approximate concentration of 10^6^ cells.ml^−1^.

An aliquot of the blood sample was applied onto a poly-L-lysine coated glass slide (VWR International BVBA, Leuven, Belgium), smeared and air dried for about 2 h. The slides were then fixed in 100 % grade methanol for few minutes, air dried and kept at 4°C until use. The remaining blood, head kidney or spleen leucocytes were mixed with latex beads (Fluoresbrite® YG Carboxylate Microspheres 1.0 μm, Polysciences Europe GmbH, Germany) at a concentration of 5 × 10^7^ beads.ml^−1^cells, to get an approximate concentration of 50 beads per cell. They were incubated at 14°C for 4 h, after which each sample was applied onto a poly-L-lysine coated glass slide, smeared, dried and preserved using the same procedure as mentioned above.

### **Tissue fixation and paraffin section**

Different tissues and organs - dorsal skin, gill, peritoneal tissue, head kidney, trunk kidney, spleen, swim bladder, liver, pyloric caeca, intestine, rectum, heart and ovary - were sampled and fixed overnight in 4 % paraformaldehyde prepared in phosphate buffer saline (PBS, pH 7.4) treated with 0.1 % diethylpyrocarbonate (Sigma). Standard histological procedures were adopted to process these samples and embed them in paraffin (Paraplast® Tissue Embedding Media, Pennsylvania, USA). Later the paraffin-blocked tissues were used to prepare 4‐5 μm sections employing a microtome (Shandon Finesse, Thermo Scientific, Barrington, USA) and these sections were then mounted on poly-L-lysine coated slides. The slides were subsequently incubated overnight at 50°C and kept at room temperature until they were used in IHC analysis.

### **Specificity of the anti-Pis1 antibody**

The specificity of the anti-Pis1 antibody was confirmed by Western blot analysis using synthetic piscidins and tissue extracts as test samples, following the procedure reported by Corrales et al. [23]. In tissue sections, the antibody specificity was determined by incubating the serial section samples from the fry stage of cod in different preparations of primary antibody. These preparations were: (i) untreated anti-Pis1 antibody (the positive assay), (ii) antibody pre-incubated with the cod Pis1 peptide, (iii) dilution buffer (1.5 % bovine serum albumin (BSA) in 0.1 M PBS) alone, (iv) antibody pre-incubated with a related peptide (cod Pis2) or (v) a non-related peptide (cecropin P1) to check cross-reactivity or (vi) an anti-cod galectin antibody, to detect false positive reactions in some test tissues and organs. The pre-incubation of antibody with each peptide was performed following the procedure reported by Sompuram et al. [25]. Briefly, undiluted anti-Pis1 antibody (727 μg⋅ml^-1^, according to GenScript) was mixed with an equal volume of each peptide solution, maintaining a ratio of 1:2 for the concentration of the antibody and the peptide, and incubated at 37°C for 45 min. The pre-absorbed antibody was later diluted in dilution buffer to make the final concentration of ~14.5 μg⋅ml^−1^ before being used for IHC.

### **Immunohistochemistry**

IHC was performed on both smear and section samples, using procedures of Mulero et al. [22], but with some modifications. Briefly, paraffin sections prepared as described under tissue fixation were dewaxed with xylene followed by rehydration through decreasing gradients of ethyl alcohol. In the standardization steps of IHC analyses, two different antigen retrieval (AR) solutions, which are widely used for antigen retrieval in IHC studies [26], were evaluated - citrate buffer (10 mM sodium citrate, 0.05 % Tween 20, pH 6.0) and Tris-EDTA buffer (10 mM Tris-Base, 1 mM EDTA solution, pH ~9), at high (autoclave at 100°C, 10 min) and low (water bath at 65°C, 1 h) temperatures. Immunostaining of Pis1 in the tissue sections retrieved with citrate buffer at both low and high temperatures was faint, while the sections retrieved with Tris-EDTA buffer gave stronger Pis1 immunoreactivity and a desirable light background at high temperature. The last set of conditions was chosen for the IHC studies. The antigenic epitope of rehydrated specimens were retrieved by immersing slides in a staining dish containing Tris-EDTA buffer. The dish with the slides was autoclaved at 100°C for 10 min and subsequently sections were kept at room temperature for 20 min.

The tissue sections and all the leucocyte samples referred to previously were washed thrice for 5 min each time with de-ionized water. They were later incubated with 3 % hydrogen peroxide in 100 % methanol for 10 min at room temperature to quench endogenous peroxidase activity. This step was followed by washing each slide three times with washing buffer (1.5 % Tween 20 in 0.1 M PBS) for 10 min and incubating the slides in blocking solution (5 % BSA in 0.1 M PBS) for 1 h at room temperature. After removing the blocking solution the specimens were incubated overnight at 4°C with primary antibody at a dilution of 1:50 (~14.5 μg⋅ml^−1^, the best titer that gave good immunostaining and light background from among the different dilutions tested: 1:25, 1:50, 1:100, 1:400 and 1:800) with the dilution buffer. Sections were also incubated with a 1:50 dilution of Pis-1 pre-incubated antibody or with the dilution buffer alone (omitting primary antibody). After rinsing for 10 min with washing buffer (3 times), sections were incubated for 30 min with HRP conjugated secondary anti-rabbit antibody (Santa Cruz Biotechnology, California, USA) at 1:800 (the best titer which produced good immunostaining and light background from among those tested: 1:400, 1:800, 1:1200 and 1:2000) dilution in buffer. Following another three-step washing cycle as mentioned earlier with the buffer, the presence of the primary antibody bound to the tissue antigen was detected using liquid-stable 3,3´-diaminodbenzidine tetrahydrochloride (DAB, Sigma) solution as a dye substrate for alkaline phosphatase. The desired signal level was achieved after 3–10 min of incubation. Sections were then slightly counterstained in hematoxylin (MerckKgaA, Darmstadt, Germany), dehydrated and mounted with Eukitt mounting medium (Eukitt O. Kindler GmbH, Freiburg, Germany). The slides were observed under a binocular microscope equipped with a Leica camera (Leica Microsystem, Germany).

## **Results**

### **Anti-Pis1 antibody is specific for Atlantic cod Pis1 peptide**

**Figure 1:**
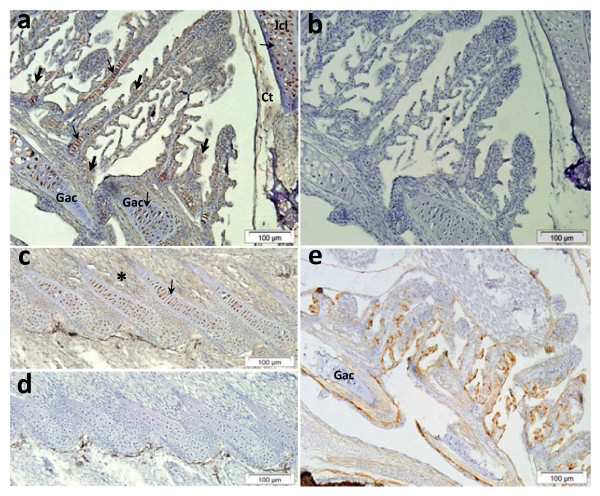
**Immunohistochemistry performed on paraffin sections of healthy Atlantic cod fry.** (**a**) Gill section treated with anti-Pis1 antibody showing immunopositive multi-granular cells (thick arrows) of the gills and a number of chondrocytes (thin arrow) in cartilaginous tissues of gill filament, gill arch (Gac), jaw cartilage (Jcl), and a weak positive reaction for the connective tissue (Ct). (**b**) Control serial section of (**a**) treated with Pis1 pre-incubated antibody indicate that all cells and tissues were not immunoreactive. (**c**) Radial cartilage of anal fin, chondrocytes (arrow) and connective tissue of muscle fibers (*) were Pis1-immunoreactive; (**d**) control serial section of (**c**) treated with Pis1 pre-incubated antibody. (**e**) Gill section treated with anti-cod galectin antibody showing immunoreactive cells scattered throughout the epithelial tissues, while all the chondrocytes were not immunostained.

Western blot analysis indicated that Pis1-antibody was immunoreactive only with Pis1 and with peptides that have molecular weight similar to that of Pis1 (Additional file 1: Figure S1). In tissue sections, we observed the positive reaction to Pis1 in various cells and tissues treated with anti-Pis1 antibody (Figure 1a and c). Pis1-immunoreactivity was also seen in serial sections and cells treated with antibody pre-incubated with either the most relevant peptide (Pis2) or the irrelevant peptide (cecropin P1; Figure not presented). On the other hand, Pis1-immunoreactivity was absent in all the control samples treated either with the antibody pre-incubated with Pis1-specific peptide or when the dilution buffer alone was used (Figure 1b and d). For all the IHC analyses that followed, negative control samples were set up by treating sections with the dilution buffer alone. In the test sample treated with the anti-cod galectin antibody, immunopositivity was found to vary depending on cells and tissues. For instance, no positive chondrocytes were seen in all cartilaginous tissues, while most of the cells in the gill epithelium were strongly immunostained compared to the anti-Pis1 antibody treated sample (Figure 1e).

### **Pis1 is produced by leucocytes, phagocytic cells and hematopoietic tissues**

Treating the samples with anti-Pis1 antibody revealed the various cell types residing in different tissues and organs of cod that are involved in Pis1 peptide production. Some immunopositive signals were also found in regions that form the cellular network of some of the tested tissues.

**Figure 2:**
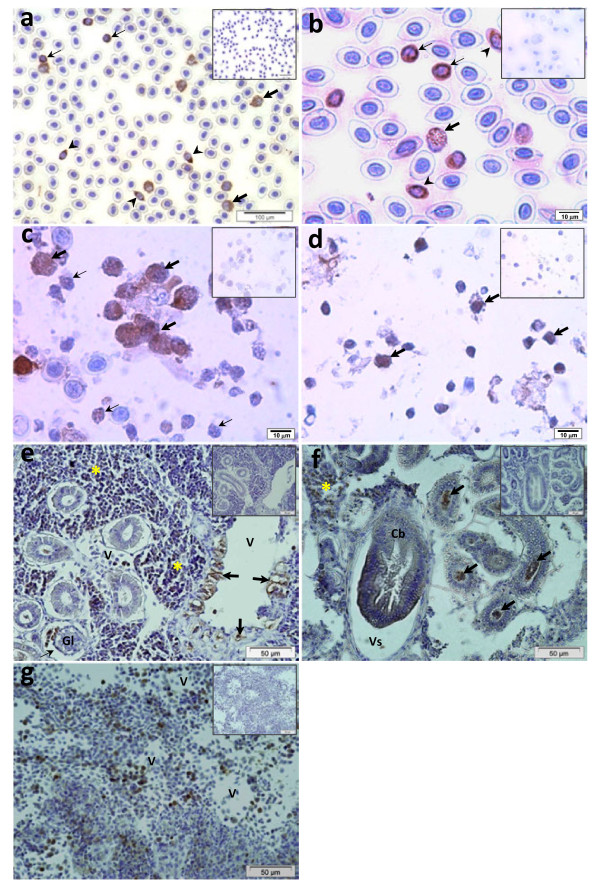
**Immunohistochemistry on Atlantic cod leucocytes and different hematopoietic tissues.** (**a**) The cytoplasm of different leucocyte types, including round cells (thin arrows), large cells with granular and lobed nuclei (thick arrows) and thrombocyte-like cells (arrow heads) were Pis1-immunoreactive (brown colour). Leucocytes incubated with latex beads (b, c and d). (**b**) Blood: some bead-containing phagocytes (thick arrows), round cells (thin arrows) and thrombocyte-like cells (arrow heads) were Pis1-immunoreactive. (**c**) Head kidney: numerous leucocytes, including phagocytes were Pis1-immunoreactive, phagocytes with intense staining had high bead uptake (thick arrows) than those with a low bead uptake (thin arrows). (**d**) Spleen: most of the leucocytes including phagocytes (arrows) were Pis1-immunoreactive. (**e**) Head kidney: numerous vascular tissue (arrows) and some cells in the hematopoietic tissues (*), glomerulus (Gl) and blood vessel (V) were Pis1-immunoreactive; note that glomerulus is surrounded by Bowman’s capsule space (unstained - thin arrow). (f) Trunk kidney: cells in hematopoietic tissue (*), cytoplasm of columnar epithelial cells of collecting tubule (Cb), cells and detached elements in the lumen of renal tubules (arrows) were Pis1-immunoreactive. (**g**) Spleen: numerous hematopoietic cells were Pis1-immunoreactive (v: blood vessel). Negative control (inset) of the cell samples and serial sections treated with dilution buffer was Pis1-immunonegative (Larger images are seen in Additional file 1: Figure S2).

Among the leucocytes isolated from the peripheral blood an oval thrombocyte-like cell, a round cell and a large cell with granular and lobed nuclei were stained positive for the anti-Pis1 antibody (Figure 2a). IHC analyses of leucocytes pre-incubated with latex beads revealed that phagocytes were able to produce Pis1. Some of the leucocytes that had phagocytosed beads were Pis1-immunoreactive (Figure 2b). Immunoreactive phagocytes were abundant in head kidney, and few in spleen cell suspensions (Figure 2c, d). Furthermore, among the head kidney leucocytes, the cells with high bead uptake exhibited a stronger Pis1-immunoreactivity than those with a lower bead uptake (Figure 2c).

In the head kidney sections, immunopositive signals were prevalent in the hematopoietic tissue, and also in the cells of glomerulus and adjacent vascular tissues (Figure 2e). In trunk kidney many Pis1-immunoreactive cells were also observed in the hematopoietic tissue, and in the cytoplasm of the columnar epithelial cells of collecting tubules and in cell debris located in the lumen of renal tubules (Figure 2f). In the spleen, numerous hematopoietic cells were also Pis1-immunoreactive, particularly those residing close to the blood vessels (Figure 2g).

### **Pis1 is produced by mucosal tissues**

**Figure 3:**
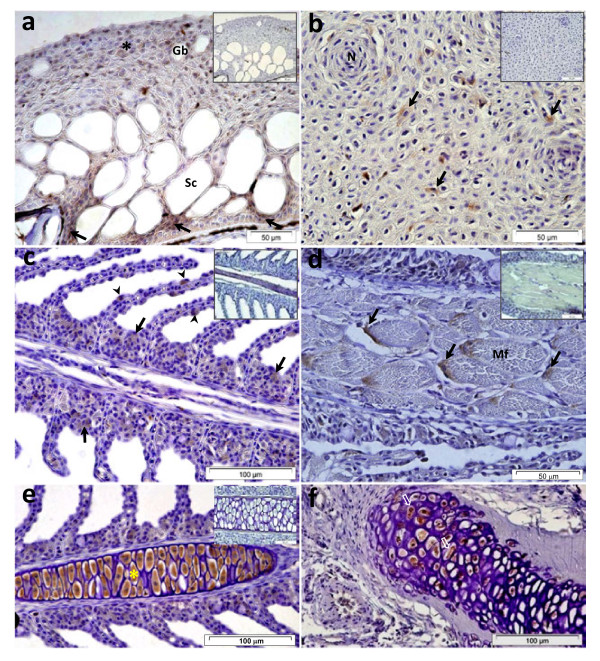
**Immunohistochemical micrographs of different tissues of Atlantic cod.** (**a**) Dorsal skin: numerous epithelial cells were Pis1-immunoreactive, but the basal cells (arrows) exhibited stronger immunoreactivity than the apical cells (Gb: goblet cell; Sc: saccular cell). (**b**) Epidermal layer of skin surrounding the eye - some Pis1-immunoreactivity was observed in the cytoplasm of epithelial cells (arrows), (N: cells of neuromast). (**c**) Gill filament: multi-granular cells of primary lamella (arrows) and epithelial cells of secondary lamella (arrow heads) were Pis1-immunoreactive. (**d**) Adductor muscle: multi-granular cells adjacent to the muscle fibers (Mf) were Pis1-immunostained. (**e**) Cartilaginous tissue of the primary lamella: all the chondrocytes (*) exhibited very strong Pis1-immunoreactivity. (**f**) Cartilaginous tissue of the gill filament: only chondrocytes (open arrows) were Pis1-immunostained (positive). Negative control (inset) of the samples treated in dilution buffer was Pis1-immunonegative (Larger images are seen in Additional file 1: Figure S3).

In the dorsal skin sections, Pis1-immunoreactivity was observed in the epithelial and basal cells of the epidermal layer (Figure 3a). Some skin epidermal tissues, collected from the vicinity of eyes, were also found to be immunopositive (Figure 3b).

In the gill section, respiratory epithelial cells and multi-granular cells of primary and secondary lamellae were Pis1-immunoreactive (Figure 3c). Some multi-granular cells of the adductor muscle were also positive (Figure 3d). Interestingly, Pis1-immunoreactivity of chondrocytes was strong in the cartilaginous tissues of both filaments and gill arches (Figure 3e-f).

**Figure 4:**
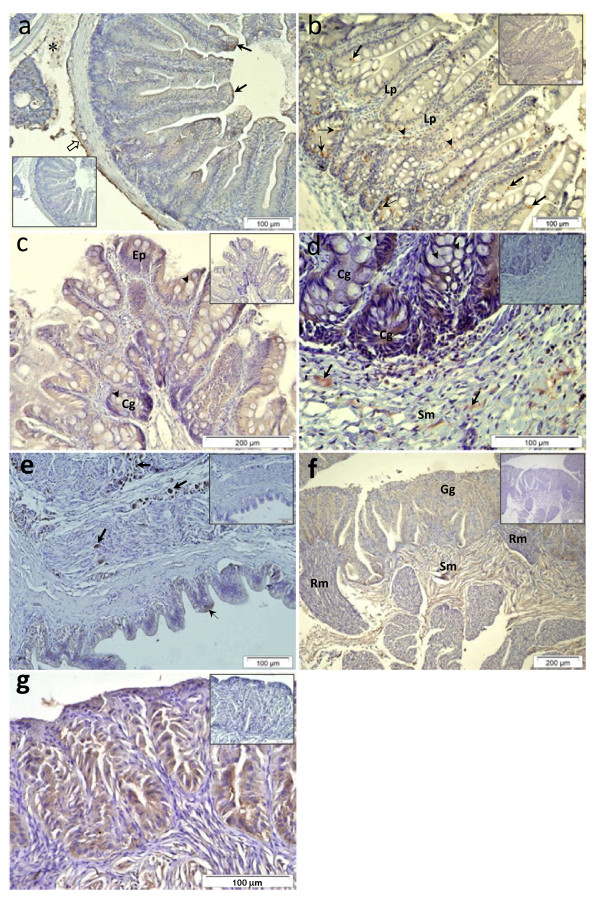
**Immunohistochemical micrographs of the gastrointestinal tract and different parts of swim bladder of Atlantic cod.** (**a**) Pyloric caeca: epithelial cells of the caecal mucosal fold (MuFo; arrows) were Pis1-immunoreactive, and some positivity was also observed in the surrounding tissues (open arrow) and between each caecal portion (*). (**b**) Proximal intestine: cells in mucosal epithelium (thick arrows) and compound glands (thin arrows), cells in lamina propria (Lp) and mucosal epithelium (arrow heads) were also Pis1-immunoreactive. (**c and d**) Rectum: cytoplasm of mucosal epithelium (Ep) and cytoplasm of columnar cells in compound glands (Cg) shown in (c) and (d), respectively were Pis1-immunoreactive, while goblet cells (arrow heads) in both mucosal epithelium and compound glands were immunonegative. Some positive multi-granular cells (arrows) in submucosa tissue (Sm) shown in (**d**) were also Pis1-positive. (**e**) A weak immunoreactivity was observed in mucosal epithelial cells (thin arrow), while a stronger positive signal was observed in the neuronal cell bodies (thick arrows). (**f**) Gas gland (Gg) and the connective tissue of the submucosa (Sm) were Pis1-immunoreactive, while a weaker positive signal was observed in the rete mirabile (Rm). (**g**) Higher magnification of gas gland showing the immunoreactivity of the gas gland cells. Negative control (inset) of the serial sections treated with dilution buffer was Pis1-immunonegative (Larger images are seen in Additional file 1: Figure S4).

In the gastrointestinal tract, Pis1-immunoreactivity was weak in the cytoplasm of epithelial cells of the caecal mucosal fold, and in some cells and connective tissues surrounding the caecae (Figure 4a). In the intestine, epithelial columnar cells, multi-granular cells in the lamina propria and submucosal tissues, and cytoplasm of columnar cells in compound glands [27] were immunostained, but goblet cells were Pis1-immunonegative (Figure 4b-d).

Mucosal epithelial cells of the swim bladder were only slightly Pis1-immunostained, while the intensity of staining was stronger in numerous neural cells of the swim bladder wall (Figure 4e). In addition, a strong positive reaction was observed in the fibroblasts, the cellular network of submucosal tissue and in the secretory cells of the gas gland, while the signal was weak in the rete mirabile (Figure 4f-g).

### **Pis1 is produced by neuronal cells, exocrine and endocrine glands**

**Figure 5:**
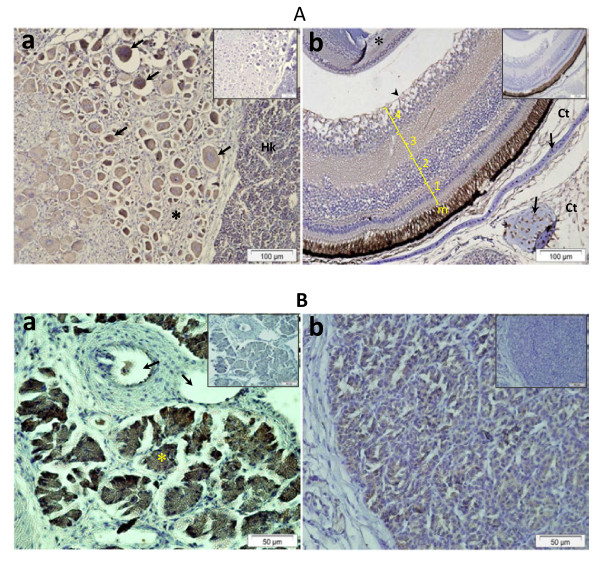
**(5A) Immunohistochemical micrographs of different neural tissues (including eye) of Atlantic cod.** (**a**) Parasympathetic ganglia: various sized neuronal cell bodies (arrows), cells and tissues in axons and dendrites (*) were Pis1-immunoreactive (Hk: head kidney’s hematopoietic tissue). (**b**) Eye: Pis1-immunoreactivity was found in various cell types and tissues –including 1: sensory cells of the retina; 2: cells and tissues of inner nuclear layer; 3: inner plexiform layer; 4: ganglion cell and nerve fiber layer, and arrowhead shows the inner limiting membrane. The chondrocytes of the hyaline cartilage (arrows), connective tissues (Ct), and tissue of the lens (*) were also immunoreactive, (m: melanocytes). (**5B**) Section of exocrine and endocrine glands of Atlantic cod. (**a**) Exocrine pancreas: the cords of exocrine acinar cells (*) showing strong intensity of Pis1-immunoreactivity; note the presence of the large hepatic veins (arrows). (**b**) Corpuscles of Stannius: Pis1-immunoreactivity was found in the cytoplasm of most secretory cells. Negative control (inset) of the serial sections treated with dilution buffer was Pis1-immunonegative. (Larger images are seen in Additional file 1: Figure S5).

The presence of Pis1 peptide was evident in the cluster of nerve cells found in the swim bladder wall as well as in the nervous system of some other organs. A large cluster of immunopositive perikarya was located on the surface of the head kidney in the parasympathetic ganglia (Figure 5A-a). In the ocular region, cartilaginous tissue, and various cells and tissues of retina - photoreceptor cells, inner nuclear layer, and nerves and nerve tissues - showed Pis1-immunoreaction (Figure 5A-b). In addition, some positive cells were also found in the choroid rete mirabile (not shown).

Examination of the sections of intestine and kidney revealed that exocrine and endocrine glands are also involved in the production of Pis1 peptide. In pancreas, most of the exocrine acinar cells exhibited strong Pis1-immunoreactivity (Figure 5B-a). In corpuscles of Stannius located on the surface tissue of both head and trunk kidneys, Pis1-immunoreactive secretory cells were found (Figure 5B-b).

### **Pis1-immunoreactivity in other tissues and organs**

**Figure 6:**
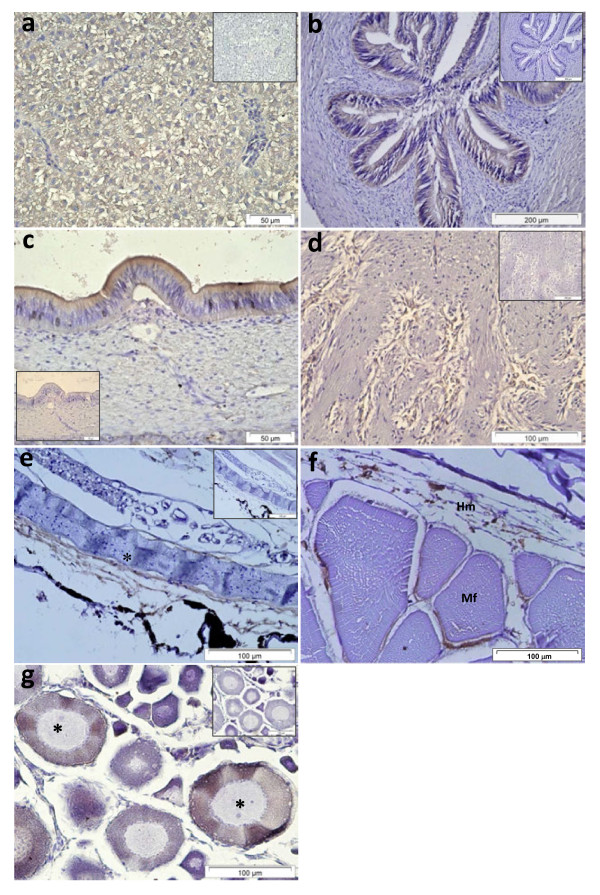
**Immunohistochemical micrographs of different tissues of Atlantic cod.** (**a**) Liver: all the hepatocyte cytoplasm and tissue that form cellular network were Pis1-immunoreactive. (**b**) Bile duct and (**c**) Gall bladder epithelium were Pis1-immunoreactive. (**d**) The tissue of bulbus arteriosus: numerous elongated fibrocytes and tissues that form cellular network were Pis1-immunoreactive. (**e**) Peritoneum: tissues that form cellular network lining a layer of mesothelium (*) were Pis1-immunostained. (**f**) Hypodermis layer (Hm) of the skin and muscle fibers (Mf): cells and connective tissues showing Pis1-positivity (**g**) Oocytes: various stages of the cells showing Pis1-immunoreactivity particularly the early or mid vitellogenic stage (*) exhibited strong positive signals. Negative control (inset) of the serial sections treated with dilution buffer was Pis1-immunonegative (Larger images are seen in Additional file 1: Figure S6).

Besides those mentioned above, some other tissues and organs were Pis1-immunoreactive. In the liver most of the hepatocytes and their cellular network were Pis1-immunostained (Figure 6a). Furthermore, the presence of this molecule was evident in the epithelial cells of gall bladder and bile ducts (Figure 6b-c). In heart, immunopositive fibroblast cells were observed in the bulbus arteriosus, while such a signal was seen neither in other parts of the heart tissue, nor in the control sample (Figure 6d).

In the peritoneum, Pis1-positivity was found in the connective tissues lining a layer of mesothelium (Figure 6e) and in cells and connective tissue of the skin hypodermal layer and around the muscle fibers (myosepta) (Figure 6f). Pis1-immunoreactivity was also noted in many stages of the oocytes; the intensity of immunostaining was stronger in the early or mid vitellogenic oocyte (Figure 6g). In addition to the gill cartilage chondrocytes mentioned earlier, other cartilaginous tissues in the radial cartilage of dorsal and anal fins, hyaline cartilage of the jaws and eyes were also Pis1-immunoreactive (see Figure 1a, c and Figure 5A-b). Further, the notochord of cod larvae was found to be immunoreactive as well (not shown).

## **Discussion**

Distribution and localisation of Pis1 peptide, a natural antibiotic, was studied in different tissues of Atlantic cod using immunohistochemistry. The specificity of the antibody was confirmed in the serial section samples, by employing anti-Pis1 antibody that was pre-incubated with either synthetic Pis1 peptide or other non-related peptides. Through Western blot analysis we have shown that the anti-Pis1 antibody of Atlantic cod does not cross react with Pis2 or Pis2b of the same species. On the other hand, similar immunostaining patterns were observed in sections treated either with the anti-Pis1antibody or pre-absorbed antibody with Pis2 peptide. The lack of cross-reactivity between anti-Pis1 antibody and Pis2 peptide could be attributed to their structural diversifications and low identity (36 % or 41 %) of Pis1 and Pis2 or Pis2b [24].

The specific antibody was used to further investigate the distribution of Pis1 peptide in fish tissues. There was sound cellular evidence on localization of Pis1 in some of the tissues, corresponding to the observations on *pis1* gene expression reported previously [24]. Various cell types, such as thrombocyte-like cells [28], multi-granular cells [29], hematopoietic cells [22] and epithelial cells from mucosal tissues [30] reacted positive to Pis1 antibody. This indicates that the molecule is probably associated with an immune function as these cells and tissues in fish are known to be involved in a dynamic immune defence network [9,29-34]. Furthermore, our observations strongly suggest that cod Pis1 activates the elimination of foreign bodies, via different mechanisms. An intracellular killing mechanism is clearly reflected through the presence of Pis1 in phagocytic cells (Figure 2). In addition, the strongly immunostained phagocytes with high bead uptake indicate that Pis1 peptide is a component of the host defence mechanism during phagocytosis. The extracellular killing mechanism, on the other hand, could be supported by the detection of peptide outside the cells of various immune organs. Antimicrobial piscidins, which are part of the defence system in many fish species, are not produced by mucosal tissues alone, but also by cells residing in the spleen and head kidney [13,22,35]. Moreover, Mulero et al. [22] have demonstrated the ability of piscidins to eliminate invading bacteria through both intracellular and extracellular killing mechanisms.

Another important observation in the present study is the discovery of the hitherto unreported information that piscidin is produced by chondrocytes of various cartilaginous tissues (Figure 1,3 and 5A). In contrast, immunohistochemical studies on other teleost species have shown that the chondrocytes did not contain piscidins [13,14,23,35]. To our knowledge, more information on teleost chondrocytes does not exist. Nevertheless, studies on mammals have shown that this cell type not only produces extracellular matrix, but also acts as a central machinery to produce AMPs and other immune factors, thus contributing to host defence mechanisms in non-vascular cartilaginous tissues [36-38]. Therefore, chondrocytes of Atlantic cod are also likely to have similar potential function, but more investigations are needed to ascertain it. A strong Pis1-immunoreactivity was also observed in the swim bladder. This organ of zebrafish was found to express several antimicrobial genes [39] and this seems to be the case as well for Atlantic cod [24,40]. As neither the genetic profiling of swim bladder nor its function in innate immune system has been described, additional studies need to be undertaken to determine the reason for the presence of AMPs or the role of AMPs in association with other established functions such as buoyancy, respiration and communication [41]. It should be noted that Silphaduang et al. [13] failed to detect piscidins in the swim bladder of hybrid striped bass, pointing to a species-dependent difference in functionality.

Atlantic cod has a unique immune system architecture compared to other vertebrates [1] and produce little or hardly any antibody upon vaccination [2,5]. Nevertheless, this fish copes with pathogen invasion [5], and its ability to protect itself could be linked to the effective innate immune components [1,3,6]. The data from this study as well as from the previous study by Ruangsri et al. [7,24] strongly suggest that Atlantic cod possesses potent antimicrobial components, particularly AMPs (piscidins), adding to the repertoire of its innate immune system.

In this study we investigated the presence of peptide in apparently healthy fish. The widespread occurrence of Pis1 suggests other possible functions of this peptide in fish, in addition to its roles against microorganisms. Pis1 may have a role in mediating cyto-protection as it was present in several tissues such as liver, bile duct and gall bladder, which are not anatomically exposed to a high pathogen pressure. Further, the high level of Pis1 peptide detected in swim bladder may signify its involvement in repair mechanisms of this tissue. In Atlantic cod, swim bladder damaged by a dramatic change of hydrostatic pressure was found to regain its function, possibly with the assistance of a membrane that lines its wall (cited by [42]). It has to be emphasized that wound healing properties of various AMPs have been reported in humans [43].

Cod Pis1 could also be a neurogenic peptide since its immunoreactivity was observed in neural tissue of some organs (Figure 4 and 5A). Little is known on AMPs and their function in nervous system of fish, however it has been demonstrated that nerve cells and or nerve tissues are never located far away from effector organs [30]. The crosstalk between nerves and immune system of the animal kingdom has long since been a topic of discussion, and it is now clear that there exists a bidirectional flow of information between the central nervous system and the immune system [44-47]. Furthermore, it has been shown that many neuropeptides and peptide hormones have similar characteristics, including antimicrobial properties [47,48]. Moreover, it is evident that together with the nervous system, the endocrine system of all animals including fish, participate in the maintenance of a steady physiologic state, homeostasis [30,47]. As Pis1 appeared in several glands (Figure 4 and 5B), this peptide may have a homeostatic role. Another fish AMP, beta-defensin (previously known as β-defensin) that was expressed highly in the pituitary and testis of orange spotted grouper is believed to maintain cellular homeostasis [49]. Apart from their antimicrobial properties, most of the hepcidins are known to maintain iron homeostasis in vertebrates including fish [50].

Involvement in osmoregulation and excretion are alternative functions which Pis1 may possess, as their presence was evident in kidney (especially trunk kidney), skin, gills, and corpuscles of Stannius of naïve fish. These organs are known to have crucial roles in the osmoregulatory and excretory mechanisms of fish besides their immune functions [30]. In addition, osmoregulatory signature of Pis1 peptide is evident from the immunoreactivity found in the rete mirabile of the swim bladder or the choroid rete mirabile of the fish eye. These unique parts are known to have key roles in oxygen secretion in the swim bladder and the eye of fish [51]. Osmoregulatory functions might also be attributed to peritoneum, hypodermis layer and myosepta where the Pis1-immunoreactivity was detected. The presence of Psoriasin1, an AMP member that belongs to the calcium-binding protein super-family was detected in peritoneal tissue of horse [52]. Pis1 peptide was also found in all stages of the oocytes, their expression being greater in the advanced developmental stages.

## **Conclusion**

It is evident that various cell types and tissues of Atlantic cod are armed with Pis1 peptide. Its constitutive presence in immune-related tissues of naïve fish suggests a significant role for this peptide in the innate immune system of Atlantic cod. In addition, the localisation of Pis1 in some of the non-immune tissues and organs confirmed our previous tissue distribution results at the molecular level [24] - suggesting other possible roles of this ubiquitous peptide and its capability to maintain homeostasis in Atlantic cod.

## **Authors’ contributions**

JR designed and performed the experiments, analyzed the data and wrote the manuscript. The experiments were overseen by JMOF, MFB, JHWMR and VK, who were also involved in the revision of the manuscript. All authors have read and approved the final manuscript.

## Supplementary Material

Additional file 1 ** Figure S1.** Tests to confirm the mono-specificity of anti-Pis1 antibody using tissue extracts from Atlantic cod and the synthetic piscidin peptide. Samples were separated on a 5–16 % acrylamide gel electrophoresis in the presence of sodium dodecyl sulfate (SDS-PAGE) running in Tris-Tricine buffer system, following the procedure of Ruangsri et al. [7]. (A) Results from coomassie blue staining. (B) Western blot analysis of piscidins and tissue extracts that were transferred onto a PVDF membrane that was blocked for 1 h and incubated overnight at 4°C with anti-Pis1 antibody. Lanes are: 1 - protein marker, 2 - leucocytes from head kidney co-incubated with latex beads for 4 h, 3 - head kidney, 4 - blood, 5 - muscle, 6 - skin, 7 - ovary, 8 - synthetic Pis2b, 9 - synthetic Pis2 and 10 - synthetic Pis1. Anti-Pis antibody was immunoreactive only with Pis1 peptide (Lane 10), while moderate to faint immunoreactivity was detected also in some tissue extracts (Lane 2 and 7) at the positions that corresponded to a molecular weight similar to that of Pis1. Figure S2. Immunohistochemical micrographs of different tissues of Atlantic cod showing absence of immunoreactivity in the control samples of (a) blood, (b) blood mixed with latex beads, (c and d) head kidney and spleen leucocytes mixed with latex beads, (e, f and g) sections of head kidney, trunk kidney and spleen treated with dilution buffer. Figure S3. Immunohistochemical micrographs of control sections treated with dilution buffer, showing immunonegative reactions in tissues of Atlantic cod. (a) Dorsal skin, (b) epidermal layer of skin surrounding eye, (c) gill filament and chondrocytes and (d) adductor muscle. Figure S4. Immunohistochemical micrographs of control sections treated with dilution buffer, showing immunonegative reactions in tissues of Atlantic cod. (a) Pyloric caeca, (b) proximal intestine, (c) mucosal epithelium of rectum, (d) compound gland and submucosa tissue of rectum, (e) swim bladder wall and mucosal epithelium , (f) gas gland and (g) higher magnification of gas gland. Figure S5. Immunohistochemical micrographs of control sections treated with dilution buffer, showing immunonegative reactions in tissues. (A-a) Parasympathetic ganglia, (A-b) eye, (B-a) exocrine pancreas and (B-b) corpuscles of Stannius. Figure S6. Immunohistochemical micrographs of control sections treated with dilution buffer, showing immunonegative reactions in tissues. (a) Liver, (b) bile duct, (c) gall bladder epithelium, (d) bulbus arteriosus, (e) peritoneum and (f) oocytes. Click here for file

## References

[B1] Star B Nederbragt AJ Jentoft S Grimholt U Malmstrom M Gregers TF Rounge TB Paulsen J Solbakken MH Sharma A The genome sequence of Atlantic cod reveals a unique immune system Nature 2011 477 207 210 10.1038/nature10342 21832995 PMC3537168

[B2] Lund V Bordal S Kjellsen O Mikkelsen H Schroder MB Comparison of antibody responses in Atlantic cod (Gadus morhuaL.) toAeromonas salmonicidaandVibrio anguillarum Dev Comp Immunol 2006 30 1145 1155 10.1016/j.dci.2006.02.004 16616955

[B3] Magnadóttir B Jónsdóttir H Helgason S Björnsson B Solem ST Pilström L Immune parameters of immunised cod (Gadus morhuaL.) Fish Shellfish Immunol 2001 11 75 89 10.1006/fsim.2000.0296 11271604

[B4] Espelid S Rødseth OM Jørgensen TØ Vaccination experiments and studies of the humoral immune responses in cod,Gadus morhuaL., to four strains of monoclonal-definedVibrio anguillarum J Fish Dis 1991 14 185 197 10.1111/j.1365-2761.1991.tb00588.x

[B5] Mikkelsen H Lund V Larsen R Seppola M Vibriosis vaccines based on various sero-subgroups ofVibrio anguillarumO2 induce specific protection in Atlantic cod (Gadus morhuaL.) juveniles Fish Shellfish Immunol 2011 30 330 339 10.1016/j.fsi.2010.11.007 21078394

[B6] Persson AC Stet RJM Pilstrom L Characterization of MHC class I and beta (2)-microglobulin sequences in Atlantic cod reveals an unusually high number of expressed class I genes Immunogenetics 1999 50 49 59 10.1007/s002510050685 10541806

[B7] Ruangsri J Fernandes JMO Brinchmann M Kiron V Antimicrobial activity in the tissues of Atlantic cod (Gadus morhuaL.) Fish Shellfish Immunol 2010 28 879 886 10.1016/j.fsi.2010.02.006 20153440

[B8] Hancock RE Peptide antibiotics Lancet 1997 349 418 422 10.1016/S0140-6736(97)80051-7 9033483

[B9] Smith VJ Fernandes MOJ Zaccone G, Marchalonis JJ, Schluter SF, Meseguer J, Kapoor BG Antimicrobial peptides of the innate immune system Fish Defenses 2009 Science Publishers, USA 241 275

[B10] Noga EJ Ullal AJ Corrales J Fernandes JMO Application of antimicrobial polypeptide host defenses to aquaculture: exploitation of downregulation and upregulation responses Comp Biochem Physiol D 2011 6 44 54 10.1016/j.cbd.2010.06.001 20584633

[B11] Sun BJ Xie HX Song Y Nie P Gene structure of an antimicrobial peptide from mandarin fish,Siniperca chuatsi(Basilewsky), suggests that moronecidins and pleurocidins belong in one family: the piscidins J Fish Dis 2007 30 335 343 10.1111/j.1365-2761.2007.00789.x 17498177

[B12] Salerno G Parrinello N Roch P Cammarata M cDNA sequence and tissue expression of an antimicrobial peptide, dicentracin; a new component of the moronecidin family isolated from head kidney leukocytes of sea bass,Dicentrarchus labrax Comp Biochem Physiol B 2007 146 521 529 10.1016/j.cbpb.2006.12.007 17292649

[B13] Silphaduang U Colorni A Noga EJ Evidence for widespread distribution of piscidin antimicrobial peptides in teleost fish Dis Aquat Org 2006 72 241 252 17190202 10.3354/dao072241

[B14] Silphaduang U Noga EJ Peptide antibiotics in mast cells of fish Nature 2001 414 268 269 10.1038/35104690 11713517

[B15] Yin ZX He W Chen WJ Yan JH Yang JN Chan SM He JG Cloning, expression and antimicrobial activity of an antimicrobial peptide, epinecidin-1, from the orange-spotted grouper,Epinephelus coioides Aquaculture 2006 253 204 211 10.1016/j.aquaculture.2005.10.002

[B16] Noga EJ Silphaduang U Park NG Seo JK Stephenson J Kozowicz S Piscidin 4, a novel member of the piscidin family of antimicrobial peptides Comp Biochem Physiol B 2009 152 299 305 19266617 10.1016/j.cbpb.2008.12.018

[B17] Fernandes JMO Ruangsri J Kiron V Atlantic cod piscidin and its diversification through positive selection PLoS One 2010 5 e9501 10.1371/journal.pone.0009501 20209162 PMC2830478

[B18] Lauth X Shike H Burns JC Westerman ME Ostland VE Carlberg JM Van Olst JC Nizet V Taylor SW Shimizu C Discovery and characterization of two isoforms of moronecidin, a novel antimicrobial peptide from hybrid striped bass J Biol Chem 2002 277 5030 5039 10.1074/jbc.M109173200 11739390

[B19] Chinchar VG Bryan L Silphadaung U Noga E Wade D Rollins-Smith L Inactivation of viruses infecting ectothermic animals by amphibian and piscine antimicrobial peptides Virology 2004 323 268 275 10.1016/j.virol.2004.02.029 15193922

[B20] Sung WS Lee J Lee DG Fungicidal effect of piscidin onCandida albicans: pore formation in lipid vesicles and activity in fungal membranes Biol Pharm Bull 2008 31 1906 1910 10.1248/bpb.31.1906 18827353

[B21] Colorni A Ullal A Heinisch G Noga EJ Activity of the antimicrobial polypeptide piscidin2 against fish ectoparasites J Fish Dis 2008 31 423 432 10.1111/j.1365-2761.2008.00922.x 18471098

[B22] Mulero I Noga EJ Meseguer J Garcia-Ayala A Mulero V The antimicrobial peptides piscidins are stored in the granules of professional phagocytic granulocytes of fish and are delivered to the bacteria-containing phagosome upon phagocytosis Dev Comp Immunol 2008 32 1531 1538 10.1016/j.dci.2008.05.015 18582499

[B23] Corrales J Mulero I Mulero V Noga EJ Detection of antimicrobial peptides related to piscidin 4 in important aquacultured fish Dev Comp Immunol 2010 34 331 343 10.1016/j.dci.2009.11.004 19913049

[B24] Ruangsri J Salger SA Caipang CMA Kiron V Fernandes JMO Differential expression and biological activity of two piscidin paralogues and a novel splice variant in Atlantic cod (Gadus morhuaL.) Fish Shellfish Immunol 2012 32 396 406 10.1016/j.fsi.2011.11.022 22178249

[B25] Sompuram SR Kodela V Zhang K Ramanathan H Radcliffe G Falb P Bogen SA A novel quality control slide for quantitative immunohistochemistry testing J Histochem Cytochem 2002 50 1425 1434 10.1177/002215540205001101 12417607

[B26] Shi SR Cote RJ Taylor CR Antigen retrieval immunohistochemistry: past, present, and future J Histochem Cytochem 1997 45 327 343 10.1177/002215549704500301 9071315

[B27] Marrison MC Histology of the Atlantic cod, Gadus morhua: an atlas 1987 Can Spec Publ Fis Aquat Sci, Part One. Digestive Tract and Associated organs. Canada

[B28] Claver JA Quaglia AIE Comparative morphology, development, and function of blood cells in nonmammalian vertebrates J Exot Pet Med 2009 18 87 97 10.1053/j.jepm.2009.04.006

[B29] Reite OB Evensen Ø Inflammatory cells of teleostean fish: a review focusing on mast cells/eosinophilic granule cells and rodlet cells Fish Shellfish Immunol 2006 20 192 208 10.1016/j.fsi.2005.01.012 15978838

[B30] Genten F Terwinghe E Danguy A Atlas of Fish Histology 2009 Science Publishers, USA

[B31] Tort L Balasch J Mackenzie S Fish immune system: a crossroads between innate and adaptive responses Immunologia 2003 23 277 286

[B32] Pakers SGM Uppalapati S Meyer W Maderson P Sell AF Kruse C: Paus R Fish matters: the relevance of fish skin biology to investigative dermatology Exp Dermatol 2010 19 313 324 10.1111/j.1600-0625.2009.01059.x 20158518

[B33] Ellis AE Innate host defense mechanisms of fish against viruses and bacteria Dev Comp Immunol 2001 25 827 839 10.1016/S0145-305X(01)00038-6 11602198

[B34] Rombout JH Abelli L Picchietti S Scapigliati G Kiron V Teleost intestinal immunology Fish Shellfish Immunol 2011 31 616 626 10.1016/j.fsi.2010.09.001 20832474

[B35] Dezfuli BS Pironi F Giari L Noga EJ Immunocytochemical localization of piscidin in mast cells of infected seabass gill Fish Shellfish Immunol 2010 28 476 482 10.1016/j.fsi.2009.12.012 20034572

[B36] Varoga D Pufe T Mentlein R Kohrs S Grohmann S Tillmann B Hassenpflug J Paulsen F Expression and regulation of antimicrobial peptides in articular joints Ann Anat 2005 187 499 508 10.1016/j.aanat.2005.03.004 16320829

[B37] Warnke PH Russo PAJ Hopfenziz M Kurz B Becker ST Sherry E Springer I Sivananthan S Antimicrobial peptide immunity protects human nasal and auricular cartilage against infection J Craniofac Surg 2010 21 198 201 10.1097/SCS.0b013e3181c50fc2 20098184

[B38] Macrory L Vaughan-Thomas A Clegg PD Innes JF An exploration of the ability of tepoxalin to ameliorate the degradation of articular cartilage in a caninein vitromodel BMC Vet Res 2009 5 25 10.1186/1746-6148-5-25 19624842 PMC2719625

[B39] Oehlers SH Flores MV Chen T Hall CJ Crosier KE Crosier PS Topographical distribution of antimicrobial genes in the zebrafish intestine Dev Comp Immunol 2011 35 385 391 10.1016/j.dci.2010.11.008 21093479

[B40] Ruangsri J Kiron V Jeppinamogeru L Fernandes JMO A novel beta defensin of Atlantic cod submitted

[B41] Nilsson S Nervous control of fish swimbladders Acta Histochem 2009 111 176 184 10.1016/j.acthis.2008.11.016 19193401

[B42] van der Kooij J Righton D Strand E Michalsen K Thorsteinsson V Svedäng H Neat FC Neuenfeldt S Life under pressure: insights from electronic data-storage tags into cod swimbladder function ICES J Mar Sci 2007 64 1293 1301 10.1093/icesjms/fsm119

[B43] Steinstraesser L Koehler T Jacobsen F Daigeler A Goertz O Langer S Kesting M Steinau H Eriksson E Hirsch T Host defense peptides in wound healing Mol Med 2008 14 528 537 18385817 10.2119/2008-00002.Steinstraesser PMC2277318

[B44] Kaplin A Bartner S Reciprocal communication between the nervous and immune systems: crosstalk, back-talk and motivational speeches Int Rev Psychiatr 2006 17 439 441 10.1080/02646830500381419 16401541

[B45] Salzet M Vieau D Day R Crosstalk between nervous and immune systems through the animal kingdom: focus on opioids Trends Neurosci 2000 23 550 555 10.1016/S0166-2236(00)01642-8 11074264

[B46] Zhang N Oppenheim JJ Crosstalk between chemokines and neuronal receptors bridges immune and nervous systems J Leukocyte Biol 2005 78 1210 1214 10.1189/jlb.0405224 16204635

[B47] Brogden KA Guthmiller JM Salzet M Zasloff M The nervous system and innate immunity: the neuropeptide connection Nat Immunol 2005 6 558 564 15908937 10.1038/ni1209

[B48] El Karim IA Linden GJ Orr DF Lundy FT Antimicrobial activity of neuropeptides against a range of micro-organisms from skin, oral, respiratory and gastrointestinal tract sites Neuroimmunology 2008 200 11 16 10.1016/j.jneuroim.2008.05.014 18603306

[B49] Jin JY Zhou L Wang Y Li Z Zhao JG Zhang QY Gui JF Antibacterial and antiviral roles of a fish beta-defensin expressed both in pituitary and testis PLoS One 2010 5 e12883 10.1371/journal.pone.0012883 21188147 PMC3004800

[B50] Rodrigues PNS Vázquez-Dorado S Neves JV Wilson JM Dual function of fish hepcidin: Response to experimental iron overload and bacterial infection in sea bass (Dicentrarchus labrax) Dev Comp Immunol 2006 30 1156 1167 10.1016/j.dci.2006.02.005 16616368

[B51] Berenbrink M Historical reconstructions of evolving physiological complexity: O2secretion in the eye and swimbladder of fishes J Exp Biol 2007 210 1641 1652 10.1242/jeb.003319 17449830

[B52] Bruhn O Grotzinger J Cascorbi I Jung S Antimicrobial peptides and proteins of the horse-insights into a well-armed organism Vet Res 2011 42 98 10.1186/1297-9716-42-98 21888650 PMC3179947

