# Supplementary material for: Ubiquitous presence of piscidin-1 in Atlantic cod as evidenced by immunolocalisation
Source: BMC Vet Res. 2012 Jul 11;8:46. doi: 10.1186/1746-6148-8-46 (PMC3395576; doi:10.1186/1746-6148-8-46)
Supplement: Additional file 1 — Figure S1. Tests to confirm the mono-specificity of anti-Pis1 antibody using tissue extracts from Atlantic cod and the synthetic piscidin peptide. Samples were separated on a 5–16 % acrylamide gel electrophoresis in the presence of sodium dodecyl sulfate (SDS-PAGE) running in Tris-Tricine buffer system, following the procedure of Ruangsri et al. [7]. (A) Results from coomassie blue staining. (B) Western blot analysis of piscidins and tissue extracts that were transferred onto a PVDF membrane that was blocked for 1 h and incubated overnight at 4°C with anti-Pis1 antibody. Lanes are: 1 - protein marker, 2 - leucocytes from head kidney co-incubated with latex beads for 4 h, 3 - head kidney, 4 - blood, 5 - muscle, 6 - skin, 7 - ovary, 8 - synthetic Pis2b, 9 - synthetic Pis2 and 10 - synthetic Pis1. Anti-Pis antibody was immunoreactive only with Pis1 peptide (Lane 10), while moderate to faint immunoreactivity was detected also in some tissue extracts (Lane 2 and 7) at the positions that corresponded to a molecular weight similar to that of Pis1. Figure S2. Immunohistochemical micrographs of different tissues of Atlantic cod showing absence of immunoreactivity in the control samples of (a) blood, (b) blood mixed with latex beads, (c and d) head kidney and spleen leucocytes mixed with latex beads, (e, f and g) sections of head kidney, trunk kidney and spleen treated with dilution buffer. Figure S3. Immunohistochemical micrographs of control sections treated with dilution buffer, showing immunonegative reactions in tissues of Atlantic cod. (a) Dorsal skin, (b) epidermal layer of skin surrounding eye, (c) gill filament and chondrocytes and (d) adductor muscle. Figure S4. Immunohistochemical micrographs of control sections treated with dilution buffer, showing immunonegative reactions in tissues of Atlantic cod. (a) Pyloric caeca, (b) proximal intestine, (c) mucosal epithelium of rectum, (d) compound gland and submucosa tissue of rectum, (e) swim bladder wall and mucosal epit [file 1746-6148-8-46-S1.pptx]

## Slide 1
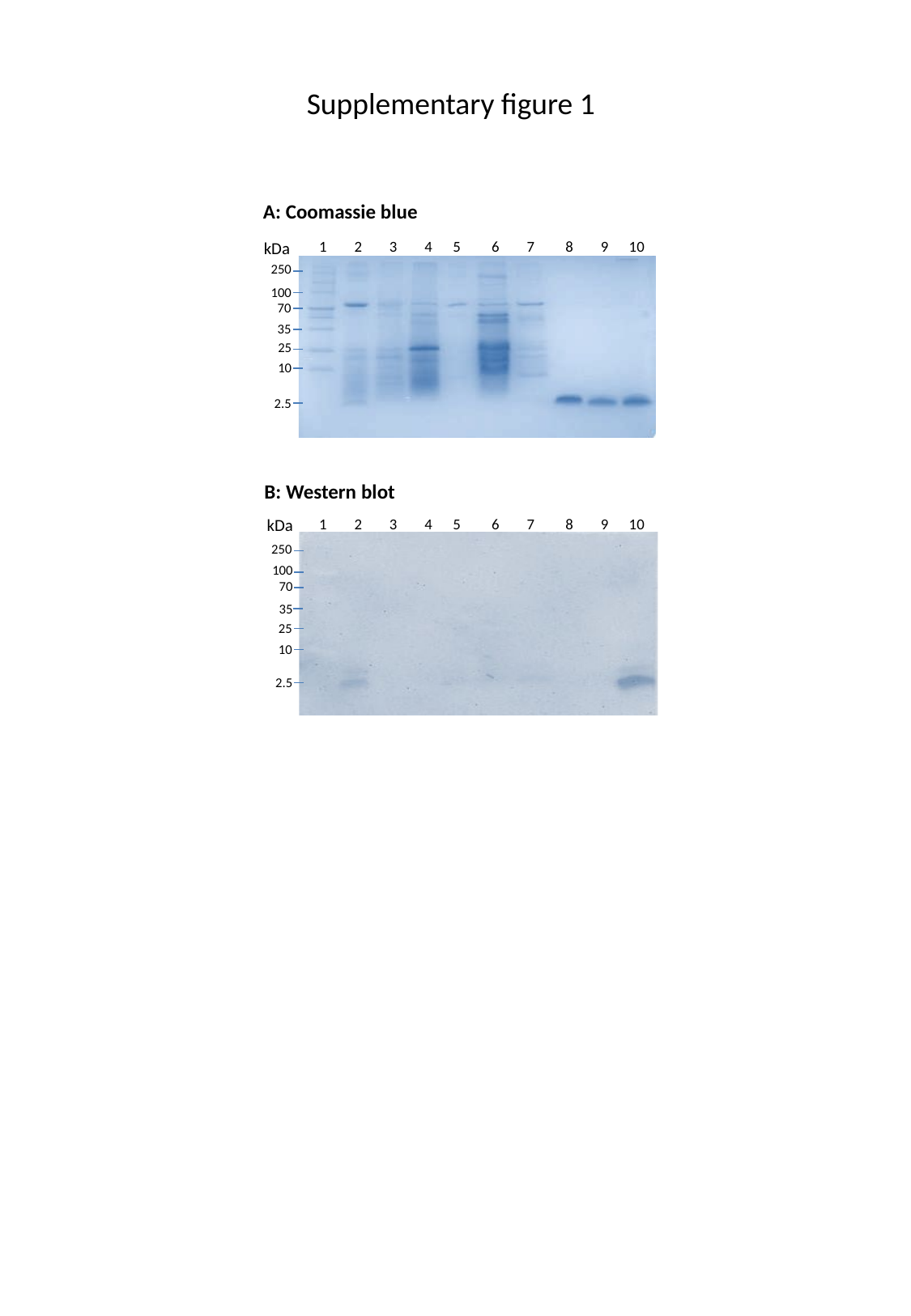

Supplementary figure 1
A: Coomassie blue
1 2 3 4 5 6 7 8 9 10
kDa
250
100
70
35
25
10
2.5
B: Western blot
kDa
1 2 3 4 5 6 7 8 9 10
250
100
70
35
25
10
2.5

## Slide 2
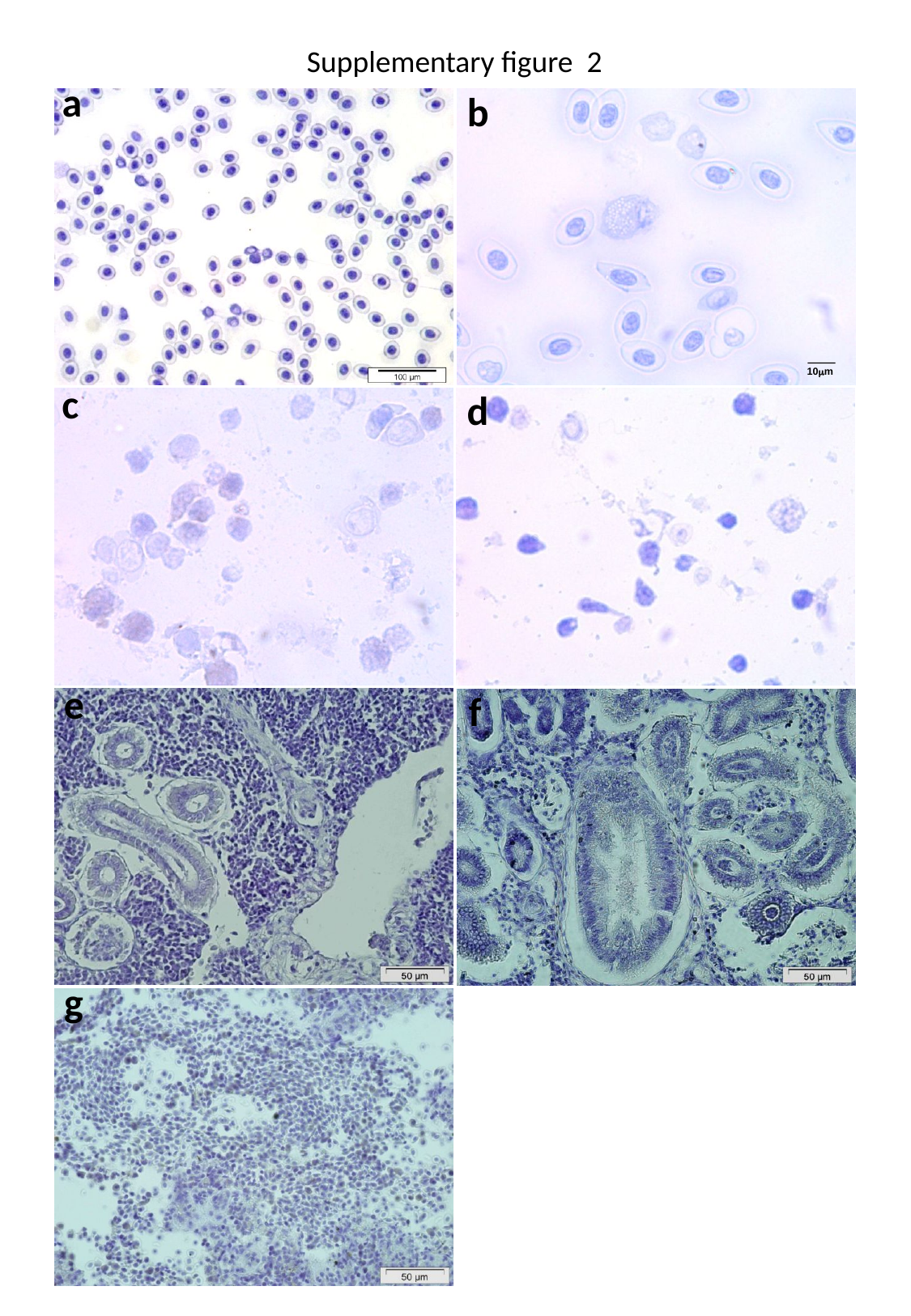

Supplementary figure 2
a
b
 10μm
c
d
e
f
g

## Slide 3
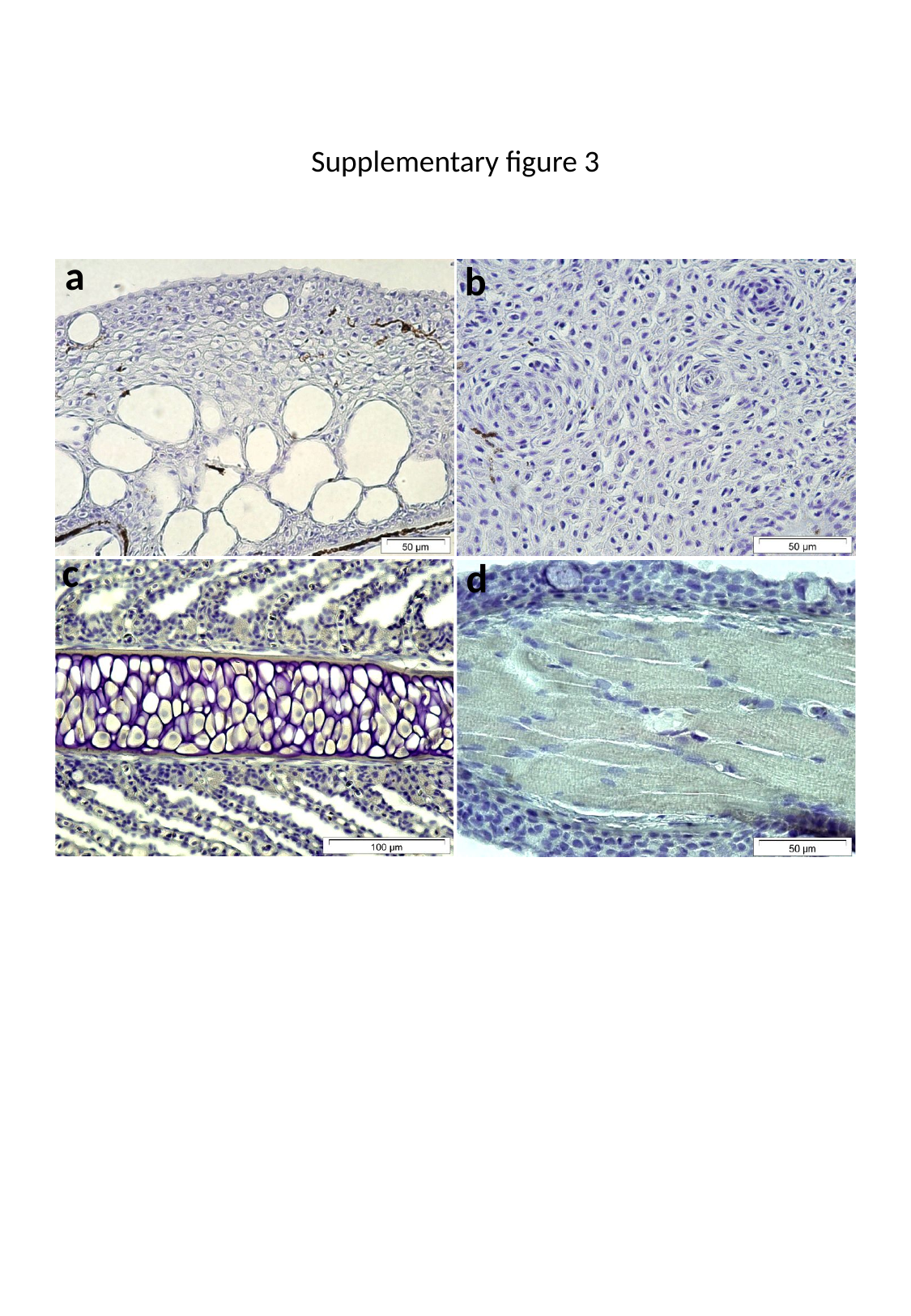

Supplementary figure 3
a
b
c
d

## Slide 4
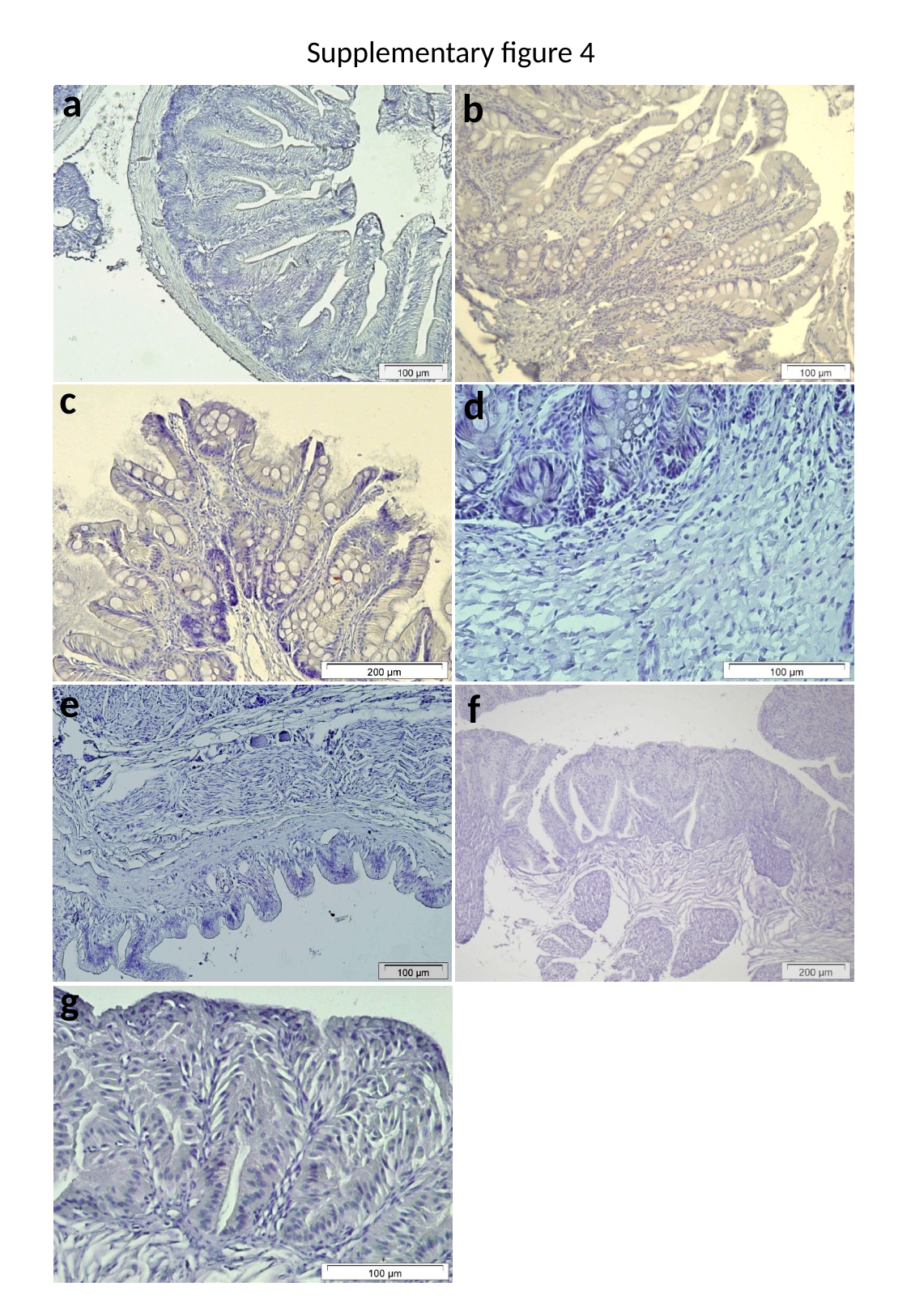

Supplementary figure 4
a
b
c
d
e
f
g

## Slide 5
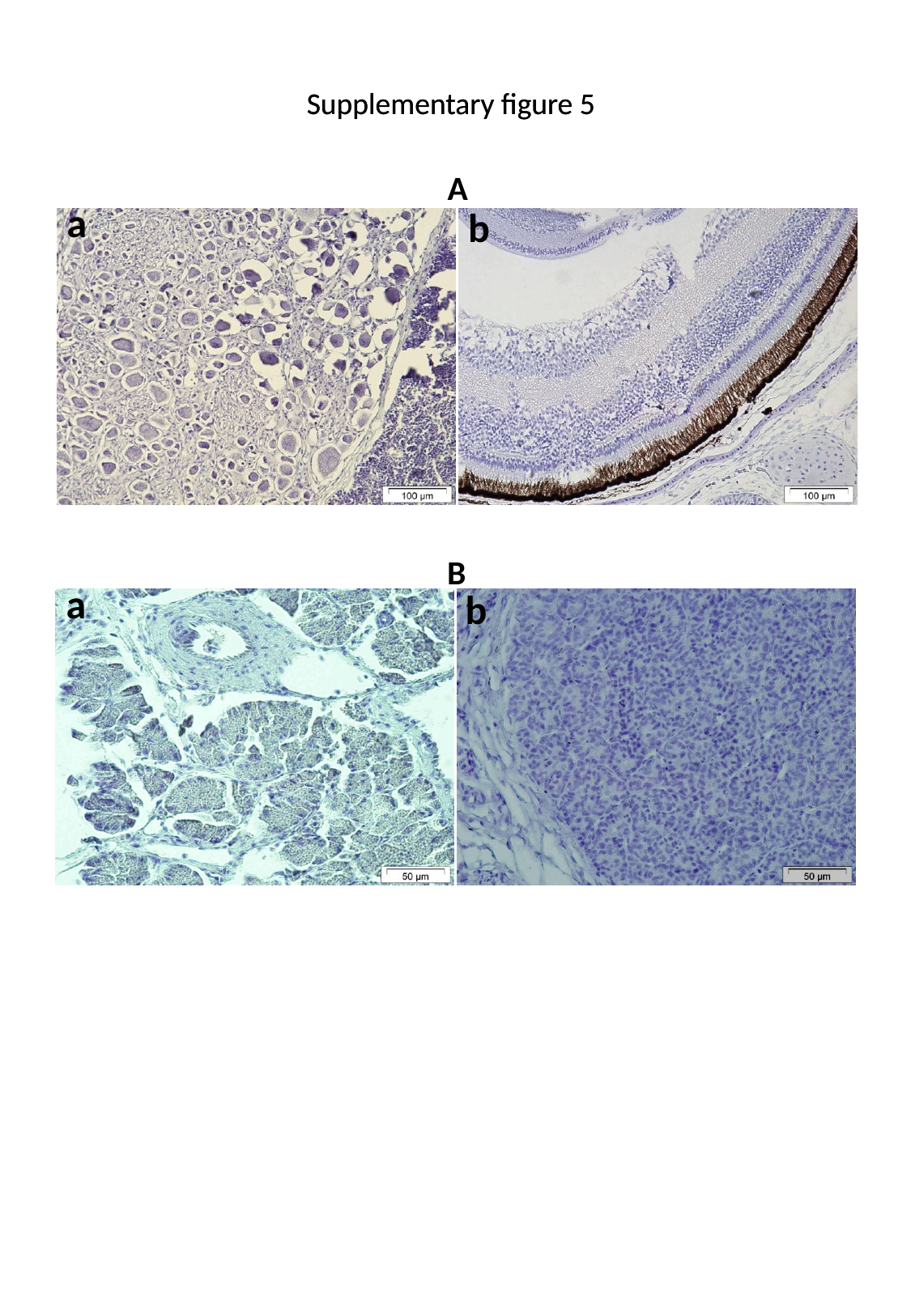

Supplementary figure 5
Supplementary figure 5
A
a
b
B
a
b

## Slide 6
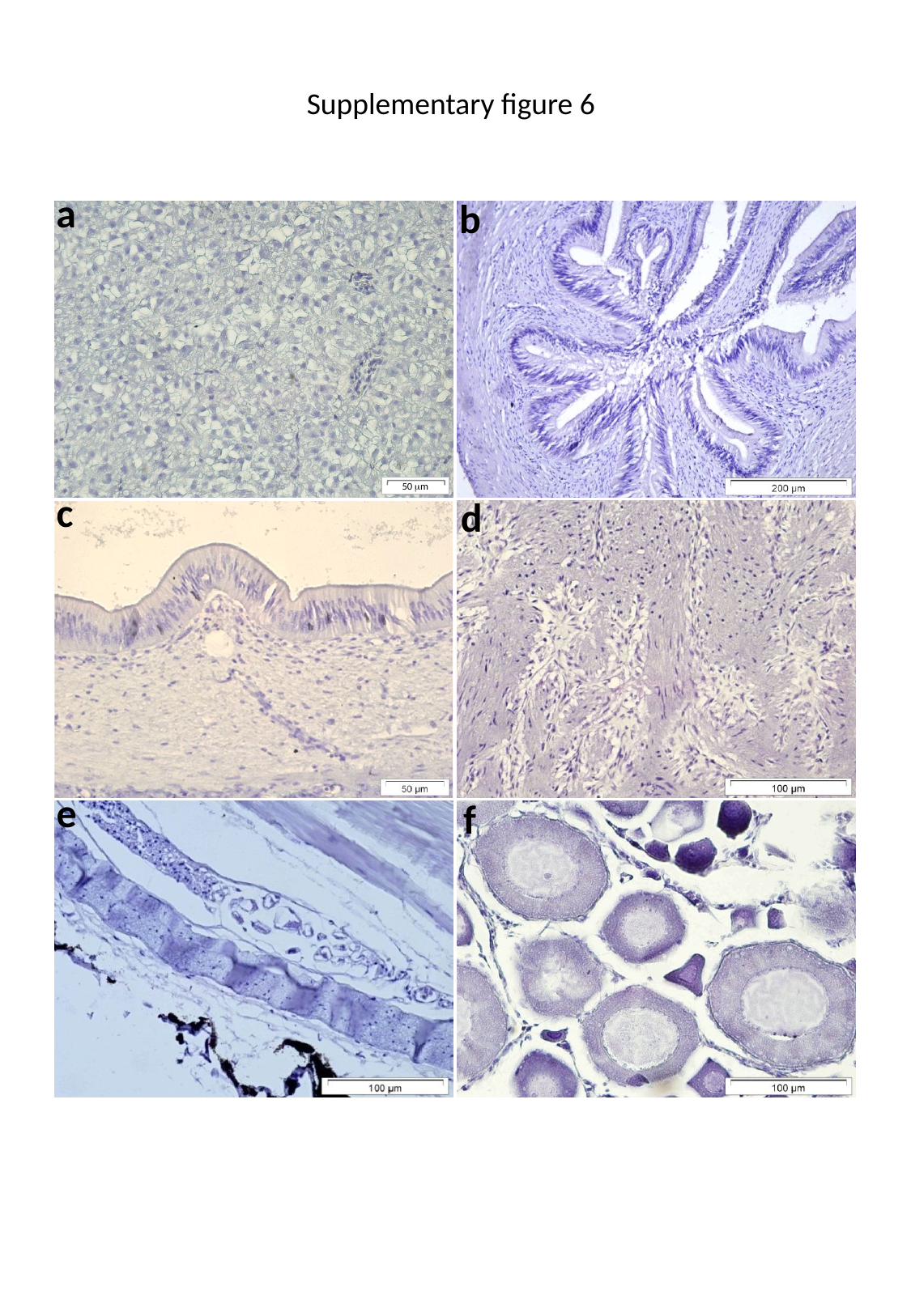

Supplementary figure 6
a
b
50 μm
c
d
e
f
